# An Interactive, Bilingual, Culturally Targeted Website About Living Kidney Donation and Transplantation for Hispanics: Development and Formative Evaluation

**DOI:** 10.2196/resprot.3838

**Published:** 2015-04-20

**Authors:** Elisa J Gordon, Joe Feinglass, Paula Carney, Daney Ramirez, Maria Olivero, Kate O'Connor, Jessica MacLean, James Brucker, Juan Carlos Caicedo

**Affiliations:** ^1^Center for Healthcare Studies and Comprehensive Transplant CenterInstitute for Public Health and MedicineNorthwestern University Feinberg School of MedicineChicago, ILUnited States; ^2^Division of General Internal Medicine and GeriatricsDepartment of MedicineNorthwestern University Feinberg School of MedicineChicago, ILUnited States; ^3^Department of Health SciencesChicago State UniversityChicago, ILUnited States; ^4^Vital Voices – PanamaPanama CityPanama; ^5^National Kidney Foundation of IllinoisChicago, ILUnited States; ^6^Grant Healthcare FoundationLake Forest, ILUnited States; ^7^Office of Public Affairs and CommunicationsNorthwestern University Feinberg School of MedicineChicago, ILUnited States; ^8^Department of Medical EducationNorthwestern University Feinberg School of MedicineChicago, ILUnited States; ^9^Comprehensive Transplant CenterNorthwestern Memorial HospitalNorthwestern University Feinberg School of MedicineChicago, ILUnited States

**Keywords:** culturally targeted, ethics, ethnic groups, eHealth intervention, Hispanic Americans, Internet, immigrants, informed consent, kidney transplantation, Latino, living donors, multimedia

## Abstract

**Background:**

As the kidney shortage continues to grow, patients on the waitlist are increasingly turning to live kidney donors for transplantation. Despite having a disproportionately higher prevalence of end-stage kidney disease (ESKD), fewer waitlisted Hispanic patients received living donor kidney transplants (LDKTs) than non-Hispanic whites in 2014. Although lack of knowledge has been identified as a barrier to living kidney donation (LKD) among Hispanics, little is known about information needs, and few bilingual educational resources provide transplant-related information addressing Hispanics’ specific concerns.

**Objective:**

This paper describes the process of developing a bilingual website targeted to the Hispanic community. The website was designed to increase knowledge about LKD among Hispanic patients with ESKD, their families, and the public, and was inspired by educational sessions targeted to Hispanic transplant patients provided by Northwestern University’s Hispanic Kidney Transplant Program.

**Methods:**

Northwestern faculty partnered with the National Kidney Foundation of Illinois for expertise in ESKD and Hispanic community partners across the Chicago area. We established a Community Advisory Board (CAB) of 10 Chicago-area Hispanic community leaders to provide insight into cultural concerns and community and patients’ needs. Website content development was informed by 9 focus groups with 76 adult Hispanic kidney transplant recipients, living kidney donors, dialysis patients, and the general Hispanic public. The website development effort was guided by community input on images, telenovela scripts, and messages. After initial development, formal usability testing was conducted with 18 adult Hispanic kidney transplant recipients, dialysis patients, and living kidney donors to identify ways to improve navigability, design, content, comprehension, and cultural sensitivity. Usability testing revealed consistently high ratings as “easy to navigate”, “informative”, and “culturally appropriate”. Bandura’s Social Cognitive Theory and Gagne’s Conditions of Learning Theory guided website design to facilitate adult learning.

**Results:**

The website, “Infórmate: Living Kidney Donation for Hispanics/Latinos” (*Infórmate Acerca de la Donación de Riñón en Vida*), includes six sections: Treatment Options, Donation: Step-by-Step, Benefits and Risks, Financial Issues, Immigrant Issues, and Cultural Beliefs and Myths. Sections host 5-10 interactive messages that summarize important points and link to detailed explanations for users interested in learning more about specific issues. The website hosts interactive videos, multimedia testimonials, telenovelas, games, and quizzes. Photographs and videos of Hispanic living donors are shown to promote pride and ownership.

**Conclusions:**

Our success in developing a website was driven by a development team with expertise in transplantation, social science, evaluation, instructional design, and Hispanic perspectives, and by a patient-centered approach toward content and design. Based on feedback from usability testing and our CAB, the website is sensitive to Hispanic cultural sensibilities. We have nearly completed a formal evaluation of the website’s impact on increasing Hispanics’ knowledge about LKD and will disseminate the website thereafter.

## Introduction

### Disparities in Kidney Transplantation for Hispanics/Latinos

The shortage of kidneys for transplantation and ethnic disparities in living kidney donation (LKD) are major public health problems [1,2]. Living donor kidney transplantation (LDKT) is considered the optimal treatment for end-stage kidney disease (ESKD) because LDKT provides shorter waiting time, longer patient and graft survival, and better quality of life than deceased donor kidney transplantation (DDKT) [3-5]. Despite having a disproportionately higher prevalence of ESKD [6-10], a smaller proportion of waitlisted Hispanics received living donor kidney transplants than non-Hispanic whites in 2014: 4% versus 10% [11]. As Hispanics are the largest and fastest growing minority group in the United States with a high prevalence of risk factors for ESKD [12], the disparity in LDKT rates is likely to increase.

Hispanics’ low rates of LDKT have been attributed to cultural beliefs, lack of knowledge, and negative attitudes about living kidney donation [13,14]. Patient and potential donor knowledge about LDKT is associated with the likelihood of having a living donor [15,16], and interventions to increase knowledge about LDKT can increase donor evaluations and actual LDKTs [17,18].

The Internet can be an excellent medium for educating underserved populations with low health literacy [19]. The “digital divide”, or lack of access to computers or the Internet, has diminished to the point where Hispanics are using the Internet at comparable rates to non-Hispanic whites (76% versus 86%) [20], suggesting that Internet-based education is an accessible medium to learn about transplantation. Efforts are increasingly being undertaken to educate patients with chronic kidney disease (CKD) through digital media [21], which have been met with general interest among patients with CKD [22].

Internet-based interventions are increasingly used for health education and behavioral change. Similarly, targeting health interventions to cultural groups is gaining greater traction [23]. Interventions that are culturally targeted address “a set of values, principles, behaviors, attitudes, policies, and structures that enable organizations and individuals to work effectively in cross-cultural situations” [24]. By using the term “culturally targeted”, our intent was to use an anthropological approach to address deep-seated cultural values commonly shared among Hispanic communities. Additionally, we recognized Hispanic communities as heterogeneous and dynamic, and that the culture of biomedicine can affect patients’ health experiences [25]. Relatedly, although preferences for the terms “Hispanic” or “Latino” vary [13,26], we use “Hispanic” herein to refer to commonly shared language and cultural values and beliefs among a heterogeneous population in the United States [27,28].

While targeting websites to cultural groups has been extensively examined in business and marketing fields [29], and shown to be effective in website performance measures (eg, ease of use) among Spanish and other European consumers [30,31], such targeting has only recently been used in Web-based health interventions to facilitate effective uptake of health messages. For example, websites have been culturally targeted to Hispanics about regional health resources in Texas [32], Turkish immigrants in the Netherlands about depression [33] or hepatitis B screening [34], and American Indian/Alaska Native youth about smoking cessation and prevention [35,36].

We created a bilingual, culturally targeted website to increase knowledge about LKD, LDKT, and kidney transplantation among Hispanic patients with ESKD, their families, and the public. The website is called “Infórmate: Inform Yourself About Living Kidney Donation For Hispanic/Latinos” (*Infórmate Acerca de la Donación de Riñón en Vida)*. The website is designed to present information needed to make informed treatment decisions. This resource is especially needed because there are few websites about kidney donation and transplantation that address specific needs of the Hispanic community or that are in Spanish [37].

This paper describes the development of *Infórmate*. We begin by presenting the theoretical approaches guiding website design and functionality. We then describe our community engagement processes and data collection efforts used to identify culturally appropriate content and design. We describe our website content and design, and usability testing, and then conclude by describing our evaluation research.

## Methods

### Website Development Process

#### Inspiration

The website was inspired by educational sessions targeted to Hispanic transplant patients that are provided by Northwestern University’s Hispanic Kidney Transplant Program (HKTP), which have been shown to increase knowledge about LKD, as previously described [38]. The Director of the HKTP and co-author (JCC) implemented the HKTP in 2006 with the intent of increasing LDKT rates in Hispanics by addressing Hispanics’ cultural beliefs, values, and information needs during the Spanish education sessions and by encouraging family involvement in the HKTP.

#### Research Team

The research team comprised a partnership between Northwestern faculty, including a medical anthropologist/ethicist with expertise in ethical issues relating to kidney transplantation and donation (EJG), health services researcher (JF), instructional design health educators (PC and JB), a Hispanic transplant surgeon (JCC), and a Hispanic research staff member (DR), and the National Kidney Foundation of Illinois (NKFI), including the former Chief Executive Officer (KO), the Hispanic community outreach staff member (MO), and the former marketing expert (JM). The NKFI was selected as a partner given their expertise in ESKD, connections to Hispanic community partners in Chicagoland, and to host the website as a neutral, unbiased information source. Two additional staff members later assisted in translation processes, for a total of five Hispanic, bilingual team members.

#### Community Advisory Board (CAB)

Leveraging the NKFI’s community connections, we established a Community Advisory Board (CAB) of 10 Chicago-area Hispanic community leaders to provide insight into cultural concerns, community needs, and patients’ needs. Hispanic community organizations represented included the Mexican Consulate, the Hispanic outreach coordinator from Gift of Hope Organ and Tissue Donor Network (the organ procurement organization serving Illinois and Northwest Indiana), Latinos por la Salud (Latinos for Health), Family Focus, Promotoras de Salud/Mano a Mano (Women Promoting Health/Hand in Hand), Juan Diego Community Center, Sinai Health Systems, Block by Block, a kidney transplant recipient, and a transplant physician researcher. The CAB was involved in several Web development and data collection activities, as discussed below.

#### Initial Website Content

The investigator team, with input from our bilingual clinical investigators including leaders of the HKTP, developed an outline of the most critical information needs of Hispanic patients and their families. All issues selected were based on the need to improve informed treatment decision making. The investigator team was careful to neither promote nor discourage living kidney donation. Thus, the website aimed to deliver information in a neutral, balanced tone with appropriate attention to the risks and uncertainties inherent in LKD to allow potential recipients and donors to make informed treatment decisions.

Our initial learning objectives addressed topics derived from ethical and legal standards of informed consent for treatment. We needed to provide information about the procedure of living donation, risks to the donor of donating, potential benefits to the donor, risks and benefits to the recipient, alternative options to the potential donor, as well as emphasizing the voluntariness of living donation. The team drew upon formal policies, for example, the Centers for Medicare and Medicaid Services (CMS) Conditions for Hospital Participation Guidelines [39] for specific informed consent content to address for living kidney donors. Members of the research team provided professional expertise in kidney disease and transplantation and contributed their experiential clinical knowledge about Hispanics’ cultural concerns about transplantation.

The investigator team grappled with several issues in the initial content development process: (1) selecting valid sources of information, (2) determining the level of detail provided in the website, and (3) defining language commensurability of health terminology in English and Spanish. These and other issues were then taken to our community partners for discussion.

#### Source Materials

We drew upon government websites, such as Organ Procurement and Transplantation Network/United Network for Organ Sharing (OPTN/UNOS), the National Institute for Diabetes and Digestive and Kidney Diseases (NIDDK), Health Resources and Services Administration (HRSA), professional resources including the American Society of Transplant Surgeons, American Society of Transplantation, the National Kidney Foundation (NKF), and peer-reviewed publications in leading transplant journals for the most accurate, current, and credible information. We did not use material from non-professional websites such as individual donor’s blogs or transplant center websites as they potentially present information in a biased fashion.

#### Level of Detail

We developed *Infórmate* to serve as a supplemental educational resource to formal transplant center education. There was tension between providing extensive information that would offer sufficient contextual understanding but might generate information overload versus limiting the amount of text provided to key ideas. To resolve the issue, we used a heuristic to guide our selection of information: including information that users would “need to know” versus information that is “nice to know.” Considering the potentially limited health literacy level of the website users, we sought to keep the website as user-friendly as possible by limiting the amount of written text to facilitate adult learning.

#### Language Commensurability of Health Terminology

We relied on terminology used by our bilingual transplant clinicians to standardize the website. Bilingual, Hispanic members of the research team engaged in forward and back translation to ensure agreement upon the alternative phrases used to convey “donor” and “recipient”. We then tested these translations with Hispanic community audiences.

We encountered challenges in creating equivalence between the English and Spanish versions of the website. For example, one goal was to educate the public about disparities in LDKT rates among Hispanics to fuel interest in learning more about treatment options for ESKD. The word “disparity” does not translate easily into Spanish; other research similarly found that this term was not well understood among some Hispanics [40]. Therefore, we used visual approaches to convey the concept of “disparity”. For example, inline display of multiple pie charts facilitated a quick and intuitive demonstration of the concept, without relying on ambiguous language (see Multimedia Appendix 1).

### Data Collection Activities

#### Focus Groups

Website development research began with 9 focus groups conducted over 3 months with 76 adult Hispanic kidney transplant recipients, living kidney donors, dialysis patients, and the general public to identify Hispanics’ information needs, cultural beliefs, and values about LKD [13]. Participants included individuals from diverse Hispanic countries, such as Mexico, Puerto Rico, and countries in Central and South America. Participants provided feedback on website names, preferred use of “Hispanic” or “Latino”, logo designs, photographs, statistical graphs and explanations, and mission statement descriptions.

#### Community Advisory Board and Community Partner Outreach

The research team held a community event at the NKFI office suite for Hispanic donors, recipients, and dialysis patients from NKFI and Northwestern’s patient lists, and members of the CAB to discuss the project goals, mission, and approach. CAB members and volunteers interacted with the research team over dinner and we used this opportunity to video-record additional LDKT recipient and donor testimonials as well as elicit additional feedback on website content and design.

For example, because the website was going to be evaluated for increases in knowledge, it needed to be designed in a way that would allow users quick and easy access to the most important learning points within each section, as identified by the research team. We asked participants to complete a brief survey that listed 20 facts about living kidney donation on paper surveys and asked community participants to rate how important each fact was for people to know, from “very important” to “moderately important” to “not important”. We drew upon the ratings provided to inform the selection of the facts listed in the “Did you know?” column that, when clicked, transport users to the website section directly addressing that issue.

Another CAB dinner meeting was also held at the NKFI office suite to screen the initial website and obtain further input for making modifications before conducting usability testing. CAB members were organized into 4 groups of 2 to 3 members and a research team member at each computer work station as they viewed specific website sections. In addition to their initial reactions, CAB members completed a survey form rating what they liked and did not like about specific website sections.

We video-recorded interviews with donors and LKDT recipients in the Transplant Division offices at Northwestern University. We asked each donor or recipient to share his or her experiences as part of a semi-structured interview that provided short video testimonials for inclusion in the website. In addition, we asked donors and LKDT recipients to complete a brief survey that profiled their experiences as a donor or LKDT recipient to be used as captions for their website videos.

#### Usability Testing

After making further changes, the website was evaluated by a third party contractor, User Centric, Inc., dba Consumer Experiences GfK Custom Research, LLC, through two waves of testing, with website modifications in between. Usability testing involved 18 adult Hispanic kidney transplant recipients, dialysis patients, and living kidney donors, in either Spanish or English, as preferred. Eligible participants were recruited through an introductory letter followed by a phone call. Testing occurred in person (n=16) or remotely (n=2) for 60 minutes, and each participant was paid US $70. Usability testing aimed to “gauge overall user experience with the *Infórmate* website; inform future design iterations in terms of the overall user experience; identify user preferences and potential problem areas; understand if the website meets users’ expectations and goals; and evaluate if the site communicates valuable information about living kidney donation” [41]. User Centric maintains a state-of-the-art laboratory setting that includes a two-way mirror and overhead displays of the participant’s computer screen to allow the research team to unobtrusively observe and listen to each participant as he or she navigated *Infórmate*. At least 3-5 members of the research team observed each participant.

Usability testing entailed free navigation of the website followed by a series of six tasks requiring participants to find specified information on the website. The learning objectives for those tasks were determined through collaboration between the research team and User Centric staff. To illustrate, one task stated: “As you continue to learn about being a living donor for a family member, you would like to learn more about how donating will impact your daily life and activities. Show me how you would search for that information”.

User Centric evaluated website usability metrics including: time needed to find sections of the website, number of clicks to find sections, and satisfaction with navigation and content. Participants’ comments about their expectations of website design were recorded. Usability data for each scenario illuminated which tasks were easier or more difficult to achieve, thereby revealing website sections that required content or design modifications.

After completing each task, participants were asked standardized survey questions to determine how our website compared to others in terms of usability. The System Usability Scale (SUS), a 10-item scale, assesses a user’s overall usability [41]. Scores range from 0 to 100, with higher scores representing greater usability. Participants were also asked, “How likely are you to recommend this device?”, which generates the Net Promoter Score (NPS), an 11-point scale about a user’s loyalty to the website. Scores range from 0 to 10, with higher scores representing greater enthusiasm for the website. For each of the six task scenarios, participants were asked:

- “How easy was it to find what you were looking for?” to assess information findability, on a scale from 1 to 5 (range 1-5), with higher scores representing greater satisfaction.
- “How satisfied are you with the information presented in this section” to assess information satisfaction, on a scale from 1 to 5 (range 1-5), with higher scores representing greater satisfaction.
- “Rate your overall experience with this website” to assess overall website experience, on a scale from 1 to 7 (range 1-7), with higher scores representing greater satisfaction.

Website usability analysis involved descriptive statistics, for example, *t* tests to assess changes in efficiency and reduction in error between the benchmark test and re-test measures of the total usability score. User Centric provided a formal report detailing usability metrics and recommendations.

To ensure that the website met high levels of quality, we designed and modified the website using the 16-item validated instrument, “Quality Assurance Rating Tool for Internet Health Sites (Version 3)” [19]. This instrument includes metrics for website developers to assess their own website’s quality such as, “Does the health Website mention the nature of audience that the site is intended for?”

## Results

### Design

Our website is called “*Infórmate: Living Kidney Donation for Hispanics/Latinos*” (*Infórmate Acerca de la Donación de Riñón en Vida*, which translates to “Inform yourself about living kidney donation”) (Informate.org). The different name in English and in Spanish derives not from translation *per se*, but from focus group feedback that the English name should directly address the point about living donation, and that the Spanish name should present an indirect message. The website was designed using Web 2.0 design, jQuery web programming, and Articulate Storyline online training development software [42] for interactive modules according to adult learning theories and instructional design principles.

### Theoretical Approaches and Applications to Content and Design

To foster cultural sensitivity and cultivate culturally appropriate user interface design, we used Resnicow’s definition of cultural sensitivity. Because few well-validated theories of health education have been applied to website design or have been used to increase understanding of organ donation, we drew upon Gagne’s Conditions of Learning Theory and Bandura’s Social Cognitive Theory to guide website design.

### Cultural Sensitivity

Our approach to website content and design entailed addressing both “surface” and “deep” cultural structures to increase cultural members’ receptivity to the intervention [23]. Surface structure refers to matters of appearance by providing images of cultural expression that can be somewhat superficial in cultural meaning. For example, we used colors throughout the website that coincide with Hispanic sensibilities, such as orange, red, and turquoise [43]. To illustrate further, when referring to lifestyle changes for living donors following donation, we showed pictures of traditional Hispanic foods. By contrast, deep structure refers to the deep-seated beliefs, values, and meanings shared by members of the cultural group. Deep structure was addressed through the content and form of content delivery such as through immersive multimedia and telenovelas. *Infórmate* accommodated both surface and deep structures to be most effective in connecting with Hispanics.

Robert Gagne’s Conditions of Learning is based on a hierarchy of intellectual skills necessary to facilitate learning [44,45]. The website content and presentation style adheres to the nine instructional design strategies that educational programs should use to ensure that learning will occur [44,46] in the following ways. The website gains participants’ attention by using culturally sensitive design and colors, and posing questions that stimulate thought. For example, the Home Page includes a rotating banner that portrays images of Hispanic individuals engaging in healthy behaviors and enjoying family interactions. Each banner photo has a text overlay designed to gain attention with a phrase related to one of the website sections. Each banner photo is also linked to the corresponding section so a viewer can easily access that content.

The website informs users of the objectives by incorporating the learning objectives used in the instructional design phase into the introductory text of each section. In the Treatment Options section, the introductory text includes: “Find answers to questions many people ask about kidney failure and treatment options for people with kidney failure: what is kidney failure, what treatment options are there for someone who has kidney failure, what are living and deceased kidney donation, how does dialysis compare to kidney transplantation and how does living donor transplantation compare to deceased donor transplantation?” Thus, users are contextually introduced to the learning objectives of the website. The website also stimulates recall of prerequisite learning where appropriate. For example, in the Immigrant Issues section, various citizenship terms are addressed in the introduction. The user has the option of reviewing definitions of these terms in an interactive exercise. The website presents the stimulus material that engages the user in learning about living kidney donation throughout the website. The website provides learning guidance by providing cognitive processing opportunities through interaction. In the Benefits and Risks section, hovering over an interactive graphic poses a question asked by kidney donors. Clicking on this section shows current medical advice regarding the question. The website elicits the performance through the use of formative assessment. In the Cultural Beliefs and Myths section, a quiz assesses the user’s knowledge of myths versus facts. After completion of the quiz, the exercise provides feedback about the performance correctness by stating whether or not participants got answers right or wrong and why, and assesses the performance by providing a score, for example, “You got 9 out of 10 questions correct!” Last, the website enhances retention and transfer by providing PDFs to download that summarize information about facts versus myths, and advantages and disadvantages of living donation versus deceased donation.

Bandura’s Social Cognitive Theory (SCT) is based on the idea that learning occurs by observing and modeling the behaviors and attitudes of others [46-48]. Instruction can be made more efficient by modeling desired behaviors to learners and by providing situations that allow learners to use or practice that behavior to improve retention. Learning occurs by enhancing a person’s (1) self-efficacy in his or her ability to engage in a behavior or understand a topic, and (2) appreciation of the benefits of that behavior or topic (by addressing knowledge, values, outcome expectations, and emotions). Efficacy beliefs can be promoted by (1) successful experiences, (2) vicarious experiences (modeling), (3) verbal encouragement, and (4) improvement in emotional states (reducing stress or negative mood) [49]. Accordingly, the website used video testimonials and telenovelas for modeling, and drag-and-drop interactive exercises with feedback to improve knowledge as applications of SCT interactive learning strategies.

### Navigation and Elements

The website hosts numerous interactive features including hover-over effects, animated drop-down lists, and hyperlinks to other sections in *Infórmate* and to other websites. *Infórmate* orients users to their location on the website by showing the *Infórmate* logo on all pages, and provides a breadcrumb trail: the path followed by the individual as he or she moves from Web page to Web page. In these and other regards, *Infórmate* expresses Hofstede’s uncertainty avoidance (the need for clear rules and direction, and for reassurance) [50]. The top of the website shows a row of photographs of Hispanic living donors and living donor recipients to convey the target population (see Multimedia Appendix 2).

**Figure 1:**
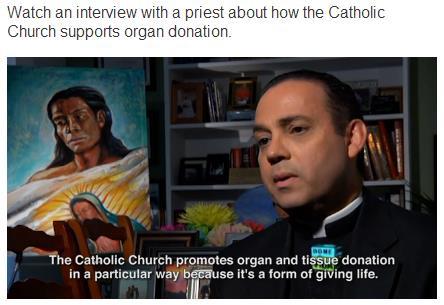
Screenshot of video of a priest is included to illustrate the authority figures represented.

Additionally, *Infórmate* hosts 19 living donor kidney recipient and living kidney donor video testimonials about their experiences of donating, life after donating, and relationships between recipient and donor. There are 8 video clips and 6 photographs of Hispanic transplant health care professionals addressing the benefits and risks of deceased donation and living donation and a priest addressing religious concerns about organ donation, thereby supporting Hofstede’s concept of high power distance and respect for authoritative figures as knowledge sources [50] (see Figure 1 and Multimedia Appendix 3).

**Figure 2:**
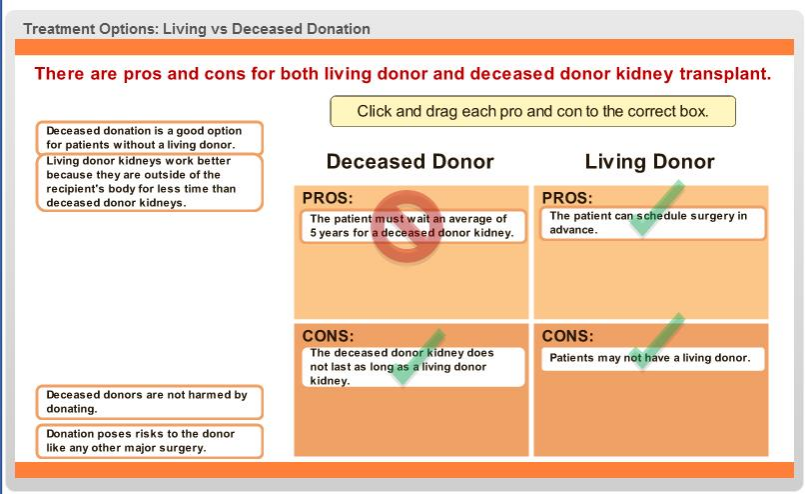
Screenshot illustrating one of the drag-and-drop games.

*Infórmate* hosts 9 interactive modules, 2 telenovelas, 3 drag-and-drop games, and a Myth versus Fact quiz about myths and misconceptions about LKD. The 100 photographs and images and 9 graphs and charts are used throughout. Free downloads of 3 fact sheets in English and Spanish as PDFs can help users remember and engage in future discussion about treatment options (eg, “Pros and Cons of Dialysis versus Kidney Transplantation”, “Pros and Cons of Living Donor and Deceased Donor Kidney Transplantation”, and “Myths and Facts about Living Kidney Donation”. Sections include videos and/or up to 10 interactive take-home messages in the “Did you know?” right-hand column that, when tapped, bring users to a detailed explanation in that section. All cross-section linking includes animated scrolling and section-expanding effects, avoiding possible navigation confusion. The website footer includes the NKFI logo and contact information (see Figure 2 and Multimedia Appendix 4).

### Structure

*Infórmate* includes six key sections: (1) *Treatment Options: Dialysis, Transplantation, and Donation*, which defines options, covers pros and cons of dialysis, transplantation, deceased donor and living donor transplantation, and addresses health disparities among Hispanics in kidney transplantation and LKDT, (2) *Benefits and Risks*, which presents the benefits, medical, and psychological risks, and potential living donor complications, as well as lifestyle changes for living donors. “Benefits” was intentionally placed before “Risks” because focus group members felt that users should know up front that there are few potential benefits to potential living donors, (3) *Donation: Step-by-Step*, which describes what tests and procedures potential donors undergo in the living donor evaluation process, (4) *Financial Issues*, which describes financial costs of transplantation and donation for donors and recipients, (5) *Immigrant Issues*, which explains the relationship between citizenship, insurance status, and being able to donate or receive a transplant, and (6) *Cultural Beliefs and Myths*, which addresses myths, misconceptions, and religious perspectives about LKD. Although our focus groups revealed that religious concerns are not a source of barriers to becoming a *living* donor, but are for becoming a *deceased* donor, we included religious perspectives about organ donation because religion plays an important role in the lives of many Hispanics [51] (see Multimedia Appendix 5).

The Home page provides a mission statement and welcome message that emphasize the focus on Hispanic communities. The About Us section includes the full mission statement, the backstory for developing the website, photos and biographies of the research team and CAB members, photos of developing the website, partners, bibliography of publications related to the study, and contact information. The use of proper titles, pictures, and an organizational chart of important people who were involved in website development in the About Us section corresponds with other research showing that Hispanics preferred business websites that display information about executives and biographical information [52].

The Resources section provides a link to a transplant hospital finder, listings and links to financial resources, pharmaceutical company programs that provide aid, websites on living kidney donation, religious perspectives on organ donation, support groups, donor and recipient testimonial videos, and a glossary.

### Text

Our focus group participants who responded to the question expressed ambivalence as to whether they preferred to be identified as “Hispanic” or “Latino”. While 44% (31/71) preferred both terms, 39% (28/71) preferred “Hispanic”, and 17% (12/71) preferred “Latino”. Thus, as most respondents selected both terms together, both are used throughout the website. All website text is available in Spanish and English, and users can effectively switch between two complete language versions of the website at any time. All text was written at a 5^th^ to 8^th^ grade reading level using the Flesch-Kincaid measure. Health literacy best practices were utilized to foster greater comprehension and simplify the presentation of written and statistical information [53-55]. For example, we used sans serif font, short sentences, active voice, plain language, second person “you” to engage viewers, posed questions as topic headers, presented frequencies with percentages, and provided diagram interpretations as titles. Further, the depth of content was layered to accommodate users with varying functional health and media literacy by providing definitions [56] (see Multimedia Appendix 6).

### Images and Telenovelas

Videos and photographs represent diverse skin tones and nationalities to provide inclusive representation [52]. *Infórmate* hosts 19 video testimonials and 20 photographs of Hispanic living kidney donors and LDKT recipients. The photos and videos assure viewers that Hispanics can be living donors. Video testimonials about Hispanic living kidney donors’ and living donor kidney recipients’ experiences of donating or receiving a living donor kidney served as role models for users. The testimonials also give users the impression that they are talking to another person about LKD. We intentionally included testimonials and conversations because focus group living donor participants reported that they preferred to observe and listen to conversations on the website rather than to read text. Testimonials are also theoretically valuable because they increase affective reactions to the message, which facilitates greater learning [57] (see Multimedia Appendix 7).

We developed two 10-minute telenovelas as a “model of” concerns commonly raised by Hispanics about living donation so that viewers can learn what to expect and become prepared to discuss those issues, and as a “model for” [58] resolving those dilemmas. Topics derived from our focus groups. Telenovelas are like soap operas and express “dramatizations of compelling stories in Spanish” [59] Telenovelas are commonly viewed among Hispanics and embed cultural values of family, community, and storytelling. Telenovelas are a culturally accepted medium for conveying health education messages among Hispanics [60], and have been shown to improve attitudes about the use of home care services [59], increase knowledge about cardiovascular disease and breast cancer [61,62], and increase behavioral intentions for mammogram screening [61].

**Figure 3:**
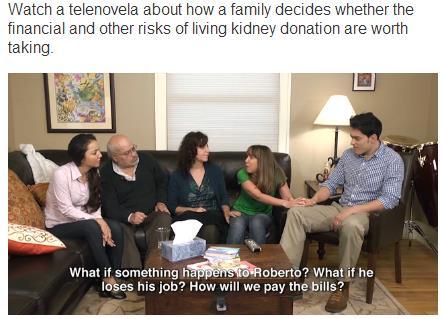
Screenshot from the Telenovela, "An Anguished Cry” (“Grito de Angustia”).

One telenovela, “*An Anguished Cry*” (“*Grito de Angustia*”), is about how a family decides whether the health risks of living kidney donation are worth taking, and addresses the concern about being able to have children after being a living kidney donor, which has contributed to reluctance to be a living kidney donor [13]. The telenovela “*The Decision*” (“*La Decisión*”), is about how a family decides whether the financial and other risks of living kidney donation are worth taking. It addresses concerns about job security and insurance coverage by discussing the financial impact on the lives of the potential living kidney donor and his or her family because living donors are out of work for 4 to 6 weeks or longer for recovery [13]. Both telenovelas were produced in Spanish with English subtitles to be consistent with the Spanish-language format of traditional telenovelas. The screenplays were written by the research team, with CAB input, and all actors were Hispanic (see Figure 3).

### Website Tone and Values

*Infórmate* conveyed a friendly tone and presented content in a polite fashion to convey respect (*respeto)* [52], by using the formal “You”, as in “*¿Sabía usted?”* (“Did you know?”), and sentimental language: “*de todo corazón*”, “*mejorarle la vida a un ser querido*”. These approaches are based on harmony in relationships, or *simpatia*, which is commonly valued in Hispanic cultures [27].

We expressed the interrelated values of collectivism (the group and group activities are prioritized over the individual [27,63]) and familism (family loyalty and solidarity is valued more than the individual or community [64]), which are commonly shared among Hispanics, by providing pictures and videos of families, images of national identity through flags representing different Latino/Hispanic countries, and using text stating “you and your family”. By showing a picture of grandparents and families on the home page, the website sought to emotionally connect [32,65].

**Figure 4:**
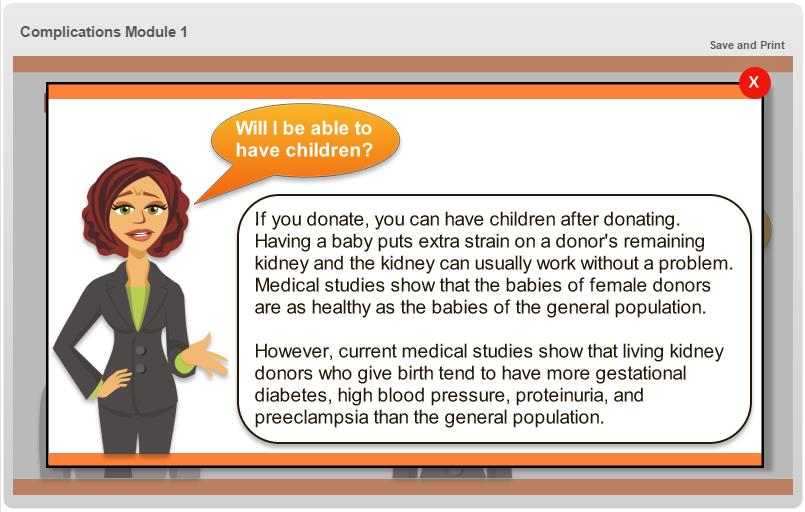
Screenshot illustrating one of the interactive modules of a woman talking about reproduction after donation.

**Figure 5:**
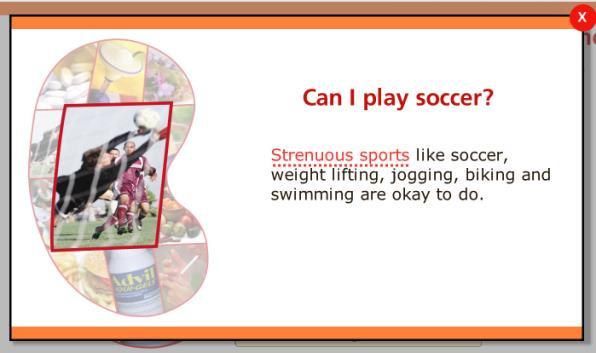
Screenshot from lifestyle changes discussion of the Benefits and Risks section, which addresses a concern about being able to play sports after donating. Screenshot also illustrates the interactive nature of this kidney-shaped module containing pictures that provide more information when selected.

We tapped into traditional Hispanic gender roles (eg, *machismo)* [27], in addressing cultural concerns. For example, concerns about the ability of both men and women to have children after donating were depicted by an interactive module, and concerns about potential living donors playing sports after donating were depicted by a photograph of soccer players (see Figures 4 and 5).

### Usability Testing

User Centric provided two reports summarizing their analyses and recommendations for website improvement after each phase of testing. Both waves of testing generated a “relatively good” SUS score compared to other websites (Table 1) [41]. The NPS scores fell into the “promoter” category representing loyal enthusiasts who will keep referring others to the site, fueling growth. Participants rated the ease of finding information highly, and participants rated their overall experience as extremely good.

Based on both reports, participants had a very positive overall impression of the *Infórmate* website. They described it as “easy to navigate and understand”, “informative and helpful”, and “very thorough”. Participants reported that they would be interested in using this website for learning more about kidney disease and treatments. Participants reported that they felt *Infórmate* was easier to use and had more interactivity than other sites they had visited, and was better than visiting multiple websites when searching for the same information. Participants found the bilingual content and topics related to immigration and financial issues to be particularly useful and unique. Both usability testing reports supported the feasibility and cultural acceptability of *Infórmate*.

Based on usability feedback, changes included increasing the size of and making more prominent the En Espaňol link used to switch from English to Spanish, creating a large play button to initiate the interactive modules, increasing the font in tables and charts, converting a complex Articulate Storyline module into a drop-down format, converting full text presentation into a drop-down format, creating cross-links between sections to facilitate ease in finding content relevant to two sections, and including animated scrolling and section-expansion to orient navigation actions. As one participant commented about the living donor testimonials:

I like it [video], these are real people, not just participants. The person who is trying to get more information, they might get more comfort knowing that they are satisfied and that it was a success.

Participant 7

**Table 1:** Characteristics and aggregated satisfaction scores of usability testing participants.

	Total n=18	Wave 1 n=12	Wave 2 n=6
Male, n (%)	8 (44)	4 (33)	4 (67)
Female, n (%)	10 (56)	8 (67)	2 (33)
Spanish, n (%)	7 (39)	5 (42)	2 (33)
English, n (%)	11 (61)	7 (58)	4 (67)
Have access to the Internet, n (%)	18 (100)	12 (100)	6 (100)
Mean System Usability Scale (SUS)		84.09	84.58
Mean Net Promoter Score (NPS)		9.91 (range 9-10)	9.67 (range 9-10)
Information findability		Range 4.11-4.75	Range 3.67-4.83
Information satisfaction		Range 4.44-5.00	Range 4.20-4.83
Overall website experience		6.64 (range 6-7)	6.0 (range 5-7)

### Health on the Net Certification (HONcode)

We also obtained Health on the Net (HONcode) certification, which is a non-governmental organization’s ethical standard for ensuring that websites offer quality health information and support transparent information to foster objectivity [66]. Our HONcode seal of certification serves to provide assurance of the quality of the website, which is consistent with research documenting Hispanics’ desire for websites to display company certifications or awards [52], and with Hofstede’s concept of uncertainty avoidance [50].

## Discussion

### Principal Results

This manuscript outlines the development process and theoretical approaches undertaken to develop a bilingual, community-focused, interactive, multimedia website about living kidney donation and transplantation targeted to Hispanics. Internet-based educational interventions can be an effective, low-cost, and private way to reach and empower many individuals [67]. The Internet can also be an optimal venue to reach underserved, low-literate populations [19], and Hispanics are increasingly using the Internet to find health-related knowledge [26]. However, even though the digital divide is shrinking, the quality, depth, and readability of transplant-related websites is poor [37,68,69]. Culturally targeted websites can reduce Hispanic health disparities by increasing knowledge [70]. Our website, *Infórmate,* was designed to redress disparities in Hispanics’ knowledge about LKD.

Given that 74% of Hispanics spoke Spanish at home in 2012 [71], our bilingual website is well suited to accommodate Hispanics of all levels of English proficiency [65]. Many of the design and formatting dimensions that we used were recommended by our Hispanic study participants and other studies’ Hispanic focus group participants’ perspectives on developing a website on cardiovascular disease prevention [40]. The sections *Financial Issues* and *Immigrant Issues* provided detailed content that addresses Hispanics’ preferences for information on immigration and insurance coverage issues [52]. Research shows that US Hispanics prefer culturally adapted website design and marketing messages [65]. Further, other research found that Internet sites involving greater use of instructional design strategies that provide feedback and foster interactivity, such as video clips, interactive modules, and animations, can increase knowledge gains and satisfaction over sites without such features [72].

Evaluation of *Infórmate* for increasing and retaining Hispanics’ knowledge about LKD using concurrent control and pretest / posttest knowledge testing is nearly complete. Upon completion, we plan to disseminate the website to transplant and dialysis clinicians throughout Illinois and nationally using evidence-based approaches [73]. In our dissemination materials (eg, pamphlets and posters), we plan to use quotes from living donors provided in response to the question, “In your opinion, how would Hispanic potential donors, recipients, and family benefit from this website?” that was posed during our video-recording session at Northwestern:

They would have access to education that currently may not exist or they may not be aware of due to the language barrier.

Female, English speaker

The website would help them in answering any questions they have and having donors talking about their experiences would help them decide.

Male, English speaker

They will all learn more on transplantation. Unfortunately, not many people in the Hispanic community know about transplants and have many myths that need to be broken.

Female, English speaker

We will evaluate our dissemination efforts by tracking metadata and phone calls to the NKFI. Our intention is for *Infórmate* to be used to initiate inquiries into LKD and to supplement transplant center education. We expect *Infórmate* to be used multiple times and that patients will use the website together with their families; this is likely to be the case among older patients with less technological savvy. Studies of Hispanics’ use of the Internet report that adult children often invite their parents to look at the website and interpret for them [32,52]. Involving family members in navigating *Infórmate* may coincide well with the integral role of Hispanic family members in decision making [74,75].

The website development process may have limitations. As the website development process was informed by Hispanics of diverse national heritages, albeit predominantly Mexican heritage, the cultural components may reflect predominantly Mexican perspectives. However, we intentionally sought to avoid using or excluding a single Hispanic/Latino cultural perspective by ensuring that our research team included Hispanics from diverse countries including Colombia, Puerto Rico, Panama, and Mexico.

### Conclusion

*Infórmate* is a bilingual Web-based educational resource about living kidney donation culturally targeted to Hispanic patients and communities. Improving Hispanics’ understanding about living kidney donation will promote autonomy and self-determination by helping to ensure that Hispanics are well informed of treatment options for ESKD.

## Multimedia Appendix 1

Diagrams used to depict the concept of disparities.

## Multimedia Appendix 2

Website banner that appears on all pages and showcases photos of Hispanic living donors and living donor recipients.

## Multimedia Appendix 3

Screenshot from one of many videos of a Hispanic transplant surgeon (JCC).

## Multimedia Appendix 4

Screenshot illustrating various interactive “Did you know?” questions used to engage the view. Different questions appear in different website sections.

## Multimedia Appendix 5

Screenshot illustrating one of the 12 questions in the Myth vs Fact game.

## Multimedia Appendix 6

Screenshot of a diagram illustrating how we provided the interpretation of the diagram’s meaning in the title, which serves as a health literacy best practice to facilitate adult learning.

## Multimedia Appendix 7

Screenshot illustrating a video of a living donor describing the financial aspects of preparing for donation.

## Abbreviations

CAB: Community Advisory Board
CKD: chronic kidney disease
CMS: Centers for Medicare and Medicaid Services
DDKT: deceased donor kidney transplantation
ESKD: end-stage kidney disease
HKTP: Hispanic Kidney Transplant Program (Northwestern University)
HONcode: Health on the Net certification
LDKT: living donor kidney transplantation
LKD: living kidney donation
NKFI: National Kidney Foundation of Illinois
NPS: Net Promoter Score
SCT: Social Cognitive Theory
SUS: System Usability Scale
