# Supplementary figures and images for: An Interactive, Bilingual, Culturally Targeted Website About Living Kidney Donation and Transplantation for Hispanics: Development and Formative Evaluation
Source: JMIR Res Protoc. 2015 Apr 20;4(2):e42. doi: 10.2196/resprot.3838 (PMC4419196; doi:10.2196/resprot.3838)

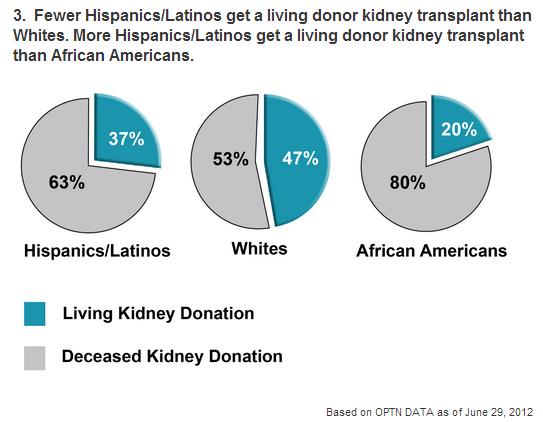

Supplement: Supplementary file 1 [file resprot_v4i2e42_app1.jpg]

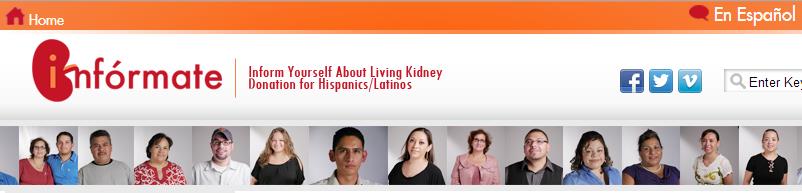

Supplement: Supplementary file 2 [file resprot_v4i2e42_app2.jpg]

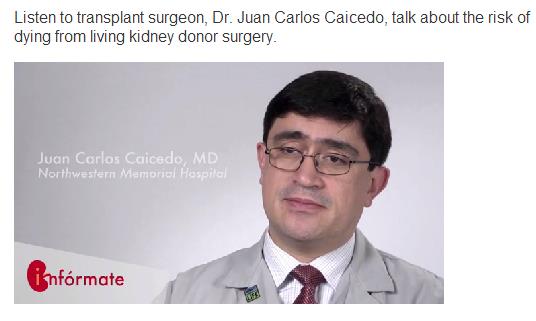

Supplement: Supplementary file 3 [file resprot_v4i2e42_app3.jpg]

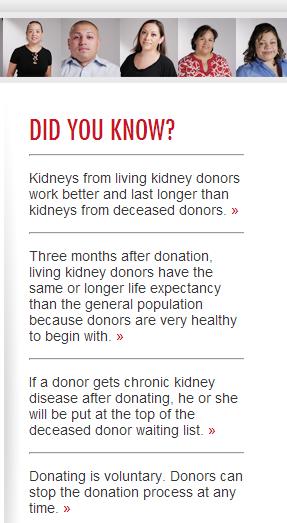

Supplement: Supplementary file 4 [file resprot_v4i2e42_app4.jpg]

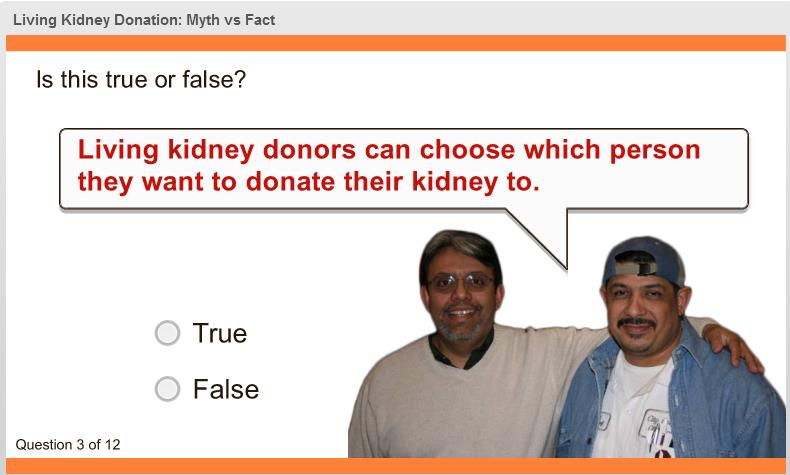

Supplement: Supplementary file 5 [file resprot_v4i2e42_app5.jpg]

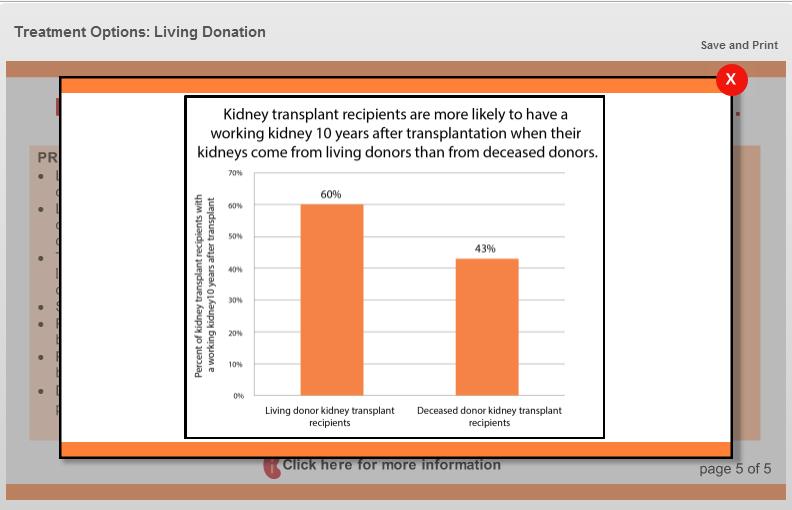

Supplement: Supplementary file 6 [file resprot_v4i2e42_app6.jpg]

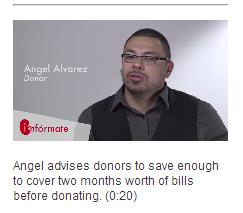

Supplement: Supplementary file 7 [file resprot_v4i2e42_app7.jpg]
